# Some Principles Governing the Development of Facial Contours in the Practice of Orthodontia

**Published:** 1893-10

**Authors:** C. S. Case


### SOME PRINCIPLES GOVERNING THE DEVELOPMENT OF FACIAL CONTOURS IN THE PRACTICE OF ORTHODONTIA.[1]

BY DR. C. S. CASE.

The writer stated his belief that we are at the beginning of a renaissance in orthodontia which will not be satisfied with the mere correction of malposed teeth, but will include as indispensable the correction of all facial deformities which have resulted from irregularities of the teeth and jaws, and the development of every æsthetic contour of the face that can be accomplished by a scientific application of force to the underlying bony structure. In the ordinary dental practice of correcting irregularities of the teeth, not enough attention has been given to facial effects; the principal aim having been to bring the teeth to a more perfect position and occlusion. While this has usually resulted in an improvement in the appearance of the face, even when the features were in repose, the development of facial contours from an æsthetic stand-point seems rather to have been a result than one of the principal aims of the operator.

In examining dental literature in this department, one is surprised to find so little said in regard to the movement of the roots of teeth, and the methods by which it may be accomplished; and in no place have I been able to find a single proposition for the outward or inward movement of the roots accompanied by a relatively slight change in the occluding position of the crowns; and

---

[1] Read before the World's Columbian Dental Congress, Chicago, August, 1893.

nothing also in regard to the movements of the roots, for the purpose of giving a more perfect contour to the face by changing the shape of the underlying bone. Dr. Farrar refers only to the movement of the entire tooth in a lateral direction. It is thus evident that the movement of the roots of teeth is a rare and somewhat modern accomplishment, and which doubtless never would have been possible under the old *régime* of regulating plates and their numerous force contrivances. Dr. Farrar thus perfectly states the only method by which a movement of the roots in the direction of the force is possible: "The secret of effecting a lateral movement of the roots of the teeth lies in relatively fixing the antagonizing ends of the crowns while the force is being applied at their necks."

The purpose of this paper is to show how this principle of force may be applied to the outward and inward, as well as to the lateral movement of the roots of the teeth; and to illustrate also the importance of this possibility, when it is observed in this operation that the bones of youth do not remain stationary to be ploughed through by the roots in a process of retrogressive metamorphosis, but that a considerable portion of the bone in which the teeth are embedded is carried with the roots in proportion as they are changed in position, thus enabling one to regulate many imperfections of the face by changing the shape and surface contour of the frame which supports and gives character to the features over all that portion which can be affected by a movement of the bones contiguous to the roots of the teeth.

The facility with which the entire intermaxillary process can be carried backward or forward under a proper application of force to the incisor teeth has been a source of surprise and pleasure to me from the time when I first attempted the operation,—less than a year ago.

I am now able to correct, with perfect certainty of success, any marked depression or protrusion of the upper lip which is mainly due to a malposition of the *roots* of the incisor teeth. Instances are frequent of comparatively perfect alignment and occlusion of the teeth, where because of the position of the roots, with a consequent abnormal depression or protrusion of the adjoining bone, considerable imperfection of features and external contour of the face is produced. Marked depression of the upper lip is often mistaken for a prognathous lower jaw, because of the lack of proper fulness in the central features of the face, which frequently affects the shape of the nose and deepens the lines on either side. For the same reason the cheek-bones will at times appear abnormally prominent, giving to the face a broad and flattened appearance; especially if the cuspids, being retarded in their eruption for the want of room, take a more lateral and prominent position. If the lower teeth are in proper relative position and the deformity caused, as is most common, by the lower incisors occluding in front of the uppers, every change desirable may be effected by an appliance attached to the superior teeth alone. As an illustration of this, I call your attention to the models in case 2, which, with others, will be fully described later.

On the other hand, if the entire superior dental arch is narrow and contracted, with a high palatal dome, the teeth long, uncrowded, and not materially affected in position by occlusion, the face will usually be long and narrow, the nose prominent, thin, and of the Roman type. In those cases the entire dental arch and alveolus should be expanded, and the force so applied and controlled as to retain the teeth in an upright position, especially in the process of carrying the anterior teeth forward, which is of vital importance in the restoration of the features of the face. The principal force, therefore, should be exerted upon the anterior superior teeth and reciprocated by rubber bands extending from the posterior part of the upper appliance to the anterior part of the lower. These bands can be made to exert almost any desired force and be worn continuously.

A large inferior dental arch with the teeth occluding outside of the superiors may be reduced in size by the extraction of a bicuspid on either side and the anterior teeth forced back to fill the space. If the chin is abnormally prominent below the incisive fossa, teeth should not be extracted from the lower, but the principal change to correct the facial deformity should be accomplished on the upper jaw.

I have abandoned all attempts to reduce a prognathous lower jaw by external pressure upon the chin. Rubber bands, extending from the upper to the lower appliance, can be made to exert all the force the patient can stand at the glenoid fossa, and tends to force the lower jaw to a more posterior position.

Protrusion of the upper lip at that point where it merges into the nasal septum and orifices may be reduced, when due to a malposition of the roots of incisor teeth alone, causing abnormal prominence of the anterior nasal spine and incisive fossa. This position is not uncommon, even when the antagonizing ends are in perfect position, and often with the production of quite a marked facial deformity.

In like manner I am able to force the anterior inferior teeth bodily forward, with the entire embedding alveolar ridge. Instances are not rare where the point of the chin, the upper lip, and the anterior superior teeth are relatively in proper position, but with inferior teeth so posteriorly placed as to produce an abnormally deep depression or curve in the lower lip along the line of the incisive fossa. By forcing the anterior inferior teeth forward with the alveolus a more æsthetic shape will be given to the chin ; a change will often produce a remarkable improvement in the general appearance of the face. The same is true, also, in forcing back the inferior incisor teeth and alveolus, when they are so anteriorly placed in relation to the point of the chin as to obliterate the graceful curve of the lower lip.

Forcing the inferior teeth and this part of the face forward is often of material aid also in the reduction of that unhappy deformity caused by a prognathous upper jaw with protruding teeth.

I believe that a large proportion of facial imperfections are due to inartistic relation of those features of the face whose form and contour are governed by the position of the teeth and the peripheral surface of the bone in which the roots are embedded. If, by force appliances attached to the crowns of the teeth of young persons, the roots and the alveolus can be forced outward or inward to any desired extent, a new field will be opened to the practitioner in orthodontia, a principal feature of which will be the correction of many deformities of the face, heretofore considered beyond the reach of orthopædic surgery. In a large proportion of those deformities which seem due to protrusion or recession of the chin, the chin is not far from its proper relative position to the forehead, the upper portion of the nose, and malar prominences, the deformity being due mainly to the relatively imperfect position of the anterior superior teeth and the adjoining bone in which they are embedded. If the crowns of these teeth are forced backward or forward to a more perfect alignment with the lower, the facial defect is only partially remedied and the real deformity far from being removed, if not increased, as it may be, by the tendency of the roots to tip in an opposite direction. But if, on the other hand, the teeth are firmly grasped by appliances so constructed that the force can be applied directly to the roots while the antagonizing ends of the crowns are fixed or controlled in their movement, it will be found that the roots as well as the immediately surrounding bone will be moved and made to take a position which will give a far more pleasing appearance to the face.

The peculiar apparatus which I use for applying force to the roots of the anterior teeth in facial contouring was first put into practical use by me December 24, 1892, and described in connection with a paper I read before the Chicago Dental Society the following February, which was published in the March number of the *Dental Review.*

In constructing an apparatus for forcing the roots and adjoining bone of the anterior teeth forward, wide German-silver banding material for the teeth should be selected, that is, five- or six-thousandths of an inch in thickness. This should be fitted to the crowns of the anterior teeth near the margins of the gum, perhaps extending beneath margins on the proximal sides. Then bars of No. 18 E. S. G. wire, slightly flattened, should be soldered to each of the bands in an upright position, and bent so as to lie along the anterior surfaces of the crowns from the apex to where the bars join the band; here they should take a direction somewhat parallel to the gum, but free from the surface to about one-eighth of an inch above its margin, at which point they should be flattened or thinned so as to be more easily bent forward and firmly clasped around a rigid bar, which is made to extend from anchorage tubes attached to the posterior teeth.

This bar, which should be very rigid, is drawn without annealing from a No. 12 extra hard German-silver wire to No. 18 E. S. G. The ends are threaded in the No. 4 hole of the Martin screw-plate, and the central portion slightly flattened in the rollers. Then it should be bent so as to rest, when in proper position, in the unclasped ends of the upright bars that have been left open to receive it. Before placing it in position the nuts should be screwed on to work at the anterior ends of the tubes.

This apparatus can be made to exert an exceedingly powerful force, but if put into practical use as it now stands the ends of the roots and adjoining bony structure would not be forced forward, notwithstanding the fact that the power is applied directly to the roots somewhat above the cervices. The crowns and the body of the roots, with a portion of the alveolus only, would be moved forward.

To complete the apparatus the fulcrum should be removed from the anterior alveolar plate, and placed so that the power can be applied between it and the ends of the roots to be moved. In other words, the crowns should be restricted or controlled in movement so that the applied force may be directed to the roots alone.

I accomplish this by a second bar, much smaller and thinner than the first but proportionately rigid, which rests in depressions in the upright pieces along the occluding ends of the teeth. The ends of the fulcrum bar are threaded and passed through tubes that are soldered to the anchorage bands on each side below the power bar tubes, with nuts which work posteriorly to the tubes.

An apparatus for reducing a prominence of the features by exerting a posterior force upon the roots and alveolus of the anterior teeth is constructed in a similar manner to that just described, with the following exception: (1) The bands should be fitted to the crowns of the incisors near their occluding ends, for the purpose of obtaining a more rigid bearing in the changed application of force. (2) The lower ends also of the upright pieces should be made to clasp the fulcrum bar. (3) The nuts should be reversed in their relative position to the tubes. (4) In moving the roots of the cuspids where required, short sections of pipe are clasped around and soft-soldered to the bars to prevent them from slipping through the clasps at the ends of the upright pieces.

In the contouring apparatus I have outlined, the force expended at the anchorage attachments is largely neutralized by the reciprocating influence of the two forces, and this reciprocation is always equal to the power used on the fulcrum bar in preventing a movement of the occluding ends of the corners. The balance of the power, which may be considerable in the general movement of the parts, must be sustained by the anchorage teeth if not further neutralized by other auxiliaries.

When the central features of the face are depressed with anterior superior teeth occluding posteriorly to the lower, accompanied by the usual real or apparent prognathous lower jaw, great reciprocating force may be beneficially obtained from the rubber bands before mentioned. These are cut from a three-eighths inch rubber regulating tube of good heft, and passed over the projecting ends of the anchorage tubes on the upper appliance to buttons on a lower appliance opposite the first bicuspids. The latter appliance may be so constructed that the force will be distributed to all the inferior teeth and indirectly to the jaw, forcing it to a more posterior position. The elastic force of the rubber bands can be made to do effective work to the full extent of their power, that would otherwise be expended upon a static anchorage neutralizing force on both the upper and lower jaws. They are useful also as an auxiliary in the reduction of a prognathous upper jaw by reversing their attachments. In these cases I also make use of the occipital force, largely for the advantage I obtain in forcing the anterior teeth farther into their sockets. Cases of prognathous upper jaw with protruding teeth are rare in which there is not abnormal prominence, at the base of the nose, of the bones that sustain the septum and wings of the nose. When the crowns alone of the anterior teeth are forced back this prominence becomes more pronounced, even though the position and appearance of the teeth and the face are improved.

In these cases, therefore, I consider it quite as important to move the roots as well as the crowns of the anterior teeth, when by so doing I find I am able to remove the entire deformity and greatly improve the general form of the face.

[Dr. Case then presented a number of models of cases showing the action of the contouring apparatus when put into practical use.]

